# Initial assessment of PNS safety for interventionalists during image-guided procedures

**DOI:** 10.1007/s10334-025-01228-4

**Published:** 2025-02-11

**Authors:** Feng Jia, Axel vom Endt, Philipp Amrein, Maximilian Frederik Russe, Heiko Rohdjess, Martino Leghissa, Maxim Zaitsev, Sebastian Littin

**Affiliations:** 1 Division of Medical Physics, Department of Diagnostic and Interventional Radiology, University Medical Center Freiburg, Faculty of Medicine, University of Freiburg, Freiburg, Germany; 2 Siemens Healthineers, Erlangen, Germany; 3 Department of Diagnostic and Interventional Radiology, University Medical Center Freiburg, Faculty of Medicine, University of Freiburg, Freiburg, Germany; 4 Siemens Healthineers, Forchheim, Germany

**Keywords:** Magnetic resonance imaging (MRI), Gradient coil, Peripheral nerve stimulation (PNS), Human body models, Interventional MRI, MRI safety

## Abstract

**Purpose:**

This study investigates peripheral nerve stimulation (PNS) safety thresholds for health professionals performing MRI procedures and the variation of arm rotations in close vicinity to the magnet bore.

**Methods:**

Employing two posable human body models, this research utilized quasi-static electromagnetic calculations and neurodynamic simulations to assess PNS thresholds. Different arm rotations are compared for standing interventionalist’s posture assuming the supine patient position, typical for medical interventions inside MRI devices.

**Results:**

This study reveals that arm rotations in standing postures result in variations in PNS thresholds. However, for all the arm poses considered, the threshold was at least 2.4 times higher compared to the patient position. Differences in PNS thresholds and electric field distributions were observed between male and female models.

**Conclusions:**

The findings suggest that when the PNS thresholds for imaging subjects are not exceeded, it is likely that a subject leaning into the bore will also not experience PNS. However, variations in PNS thresholds due to arm movements highlight the importance of considering body posture in MRI safety protocols.

## Introduction

One main challenge for speeding up the inherently slow spatial encoding in magnetic resonance imaging (MRI) is rooted in physiological factors, particularly peripheral nerve stimulation (PNS). Changes in magnetic field by switching gradient coils within MRI systems induce electric fields inside peripheral nerves. In rapid imaging sequences, such as echo-planar imaging, these electric field gradients may be sufficiently intense to stimulate peripheral nerves, potentially leading to physical sensations or muscle contractions [1–4].

Some researchers have characterized the PNS threshold of a gradient coil as the minimum change of magnetic field gradient strength ($\Delta G_{\min }$), for a given rise time, required to induce an action potential in the human body [5]. Determining the PNS thresholds for MRI gradient coils typically involves experimental studies on human subjects [4, 6–9]. These studies necessitate the construction and the implementation of the gradient system to acquire empirical data.

In order to predict PNS thresholds before the implementation of MRI gradient coils, accurate numerical calculations are essential. Recently, simulations of PNS in MRI gradient coils using human body models have been explored [10–12]. However, these models have been limited to patient positions and have not considered the proximity of other individuals, such as a parent leaning into the magnet bore during a child’s examination or health professionals during MRI-guided procedures. The PNS thresholds specific to interventional radiologists remain unknown. Based on our limited knowledge, such stimulations have not yet been observed and reported up till now. However, it is still important to investigate whether stimulations of the person performing an intervention may happen under rare conditions or can be completely excluded.

During interventional procedures, frequent arm movements of the healthcare professional incorporate both translations and rotations. The impact of translational movements on PNS thresholds has been previously analyzed in a human leg model [11]. However, PNS safety assessments for arm rotations have not been extensively studied. Furthermore, the two body models predominantly used in prior research [10–12] were not adaptable for different poses, making them unsuitable for simulating PNS thresholds during varied postures such as those in interventional procedures.

To address this limitation, this study involves simulations using two posable body models at different arm rotation angles to assess the likelihood of PNS in interventional radiologists or other individuals leaning into the bore during MRI scans.

## Methods

### Electromagnetic field calculations and neurodynamic simulations

PNS simulations were conducted as follows: initially, a magnetic vector potential generated by a gradient coil in a human body model was calculated using the Biot–Savart law. This magnetic vector potential was then employed to compute the electric field within the human body, utilizing a quasi-static magnetic solver. Subsequently, the NEURON solver [13], incorporating modified McIntyre–Richardson–Grill (MRG) neuronal models [14], was used to simulate PNS thresholds for the human body model based on the calculated electric field.

In neurodynamic simulations concerning the PNS thresholds of gradient coils, a titration process is employed [10–12]. This process entails initially adjusting the peak amplitude of the gradient coil’s gradient waveform to the precise level necessary for eliciting an action potential in each peripheral nerve. Subsequently, the PNS threshold is determined by identifying the lowest among these peak amplitudes that are still capable of inducing peripheral nerve excitation. The whole procedure combining the quasi-static magnetic solver and neurodynamic simulations was implemented in Sim4Life, using its integrated Python scripting tool (ZMT Zurich MedTech AG, Zurich, Switzerland).

### Body models

**Fig. 1:**
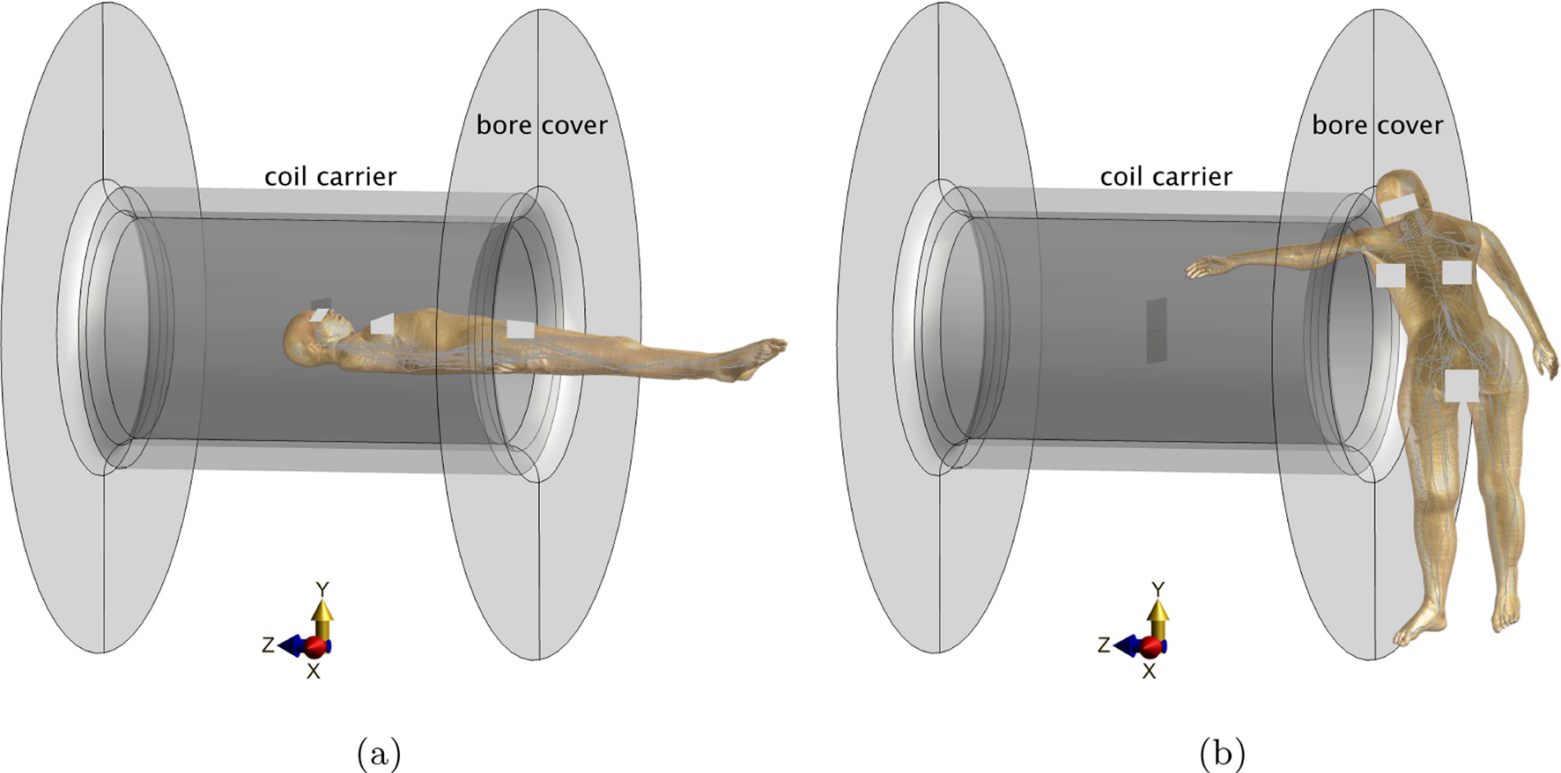
Typical position of an imaged subject (**a**) and a person performing a radiological intervention (**b**). Here, privacy-sensitive areas of the human body model have been obscured using white rectangles. The surfaces with two symmetrically flared end parts represent the bore covers of the scanner. The cylindrical ring shown denotes the gradient coil carrier, assisting in understanding the extent of the gradient coil system with respect to the arm

**Fig. 2:**
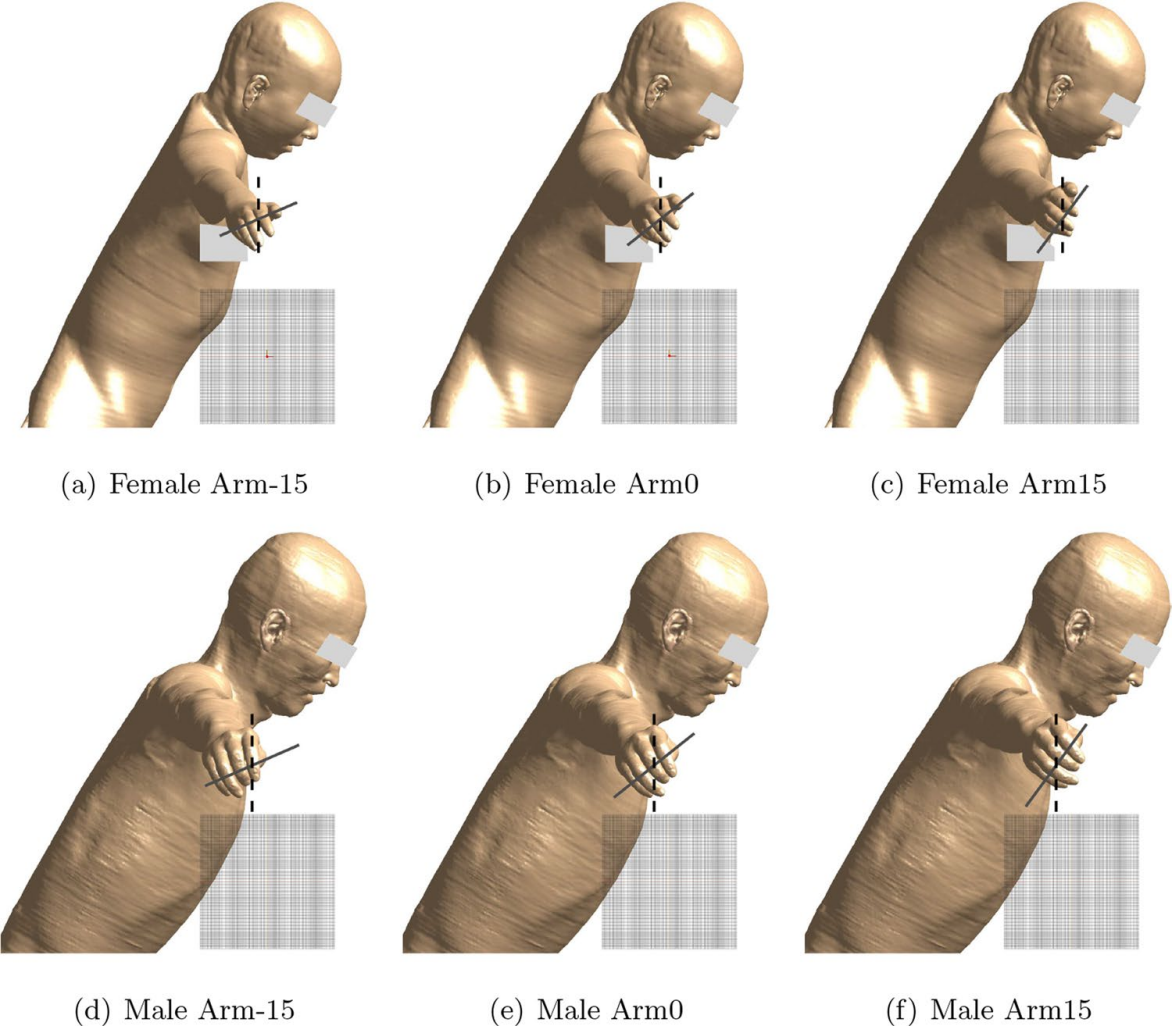
Different arm rotations of a female or male interventionalist. Here, the bodies remain as still as possible, mainly the arms are moving. Arm0 indicates that the arms are in the middle position. Arm-15 and Arm15 denote that the arms are rotated 15 degrees clockwise and counterclockwise, respectively, from the middle position. The gray solid lines indicate the arm’s rotation, while the black dashed lines serve as reference lines

For electrical field and PNS simulations, two human body models, Yoon-Sun V 4.0 (female) [15] and Jeduk V 4.0 (male) [16], featuring embedded trajectories of the main peripheral nerves (IT’IS Foundation, Zurich, Switzerland), were utilized. The initial simulations replicated a typical patient position for reference, where the subject’s head was positioned within the field of view (FOV) (Fig. 1a). To simulate PNS thresholds for a healthcare professional, both models were configured in positions proximate to the scanner bore (Fig. 1b), termed interventionalist position. The models in three distinct arm positions (Fig. 2), Arm0 (neutral), Arm15 (+ 15$^{\circ }$), and Arm-15 (– 15$^{\circ }$) rotation from the neutral position, were used to assess the effects of arm movements on PNS thresholds of the interventionalist in MRI-guided procedures. All those positions were based on real-world postures advised by a board-certified interventional radiologist.

Drawing on the methodologies in Davids et al. [12], a uniform spatial resolution (hexahedral mesh size) of 1 mm was employed for both models in the electric field simulations. Tissue electrical properties were sourced from the IT’IS low-frequency (LF) database for 1 kHz [17]. All peripheral nerves in the human body models were categorized into motor and sensory types. Motor nerves conduct impulses from the central nervous system (CNS) to effectors in muscles and glands while sensory nerves conduct impulses from the receptors to the CNS. The fiber diameters of motor and sensory nerves are simply specified to be 20 $\upmu$m and 12 $\upmu$m, respectively, as used in Davids et al. [12].

### Gradient coils and gradient waveforms

Simulations utilized wire tracks from the gradient coils of the Sonata and Aera MRI scanners (Siemens Healthineers, Erlangen, Germany). Initially, Sonata gradient coils ("BG1") were used to validate our simulations in Sim4Life, positioning the two human body models in a standard imaging pose (Fig. 1a). This validation was crucial as the experimental PNS thresholds for these coils were accessible. In Davids et al.’s study [12], these coils were also referred to as "BG1". The Sonata scanner, however, with its 60 cm bore, is suboptimal for the majority of MRI-guided interventions. Thus, following validation, "Aera" gradient coils ("AG") from a 70 cm bore scanner were used to predict PNS thresholds for the interventionalist in various arm positions.

Different axes of the gradient coil were employed to calculate the PNS thresholds. For the BG1 coils, simulations were conducted for the Y-axis, X+Y combined axes, and X+Y+Z combined axes modes to allow for comparisons with experimental data. For the AG coils, simulations were performed for the X+Y+Z, X-Y+Z, X-Y-Z, and X+Y-Z combined axes modes, each using different polarity combinations.

Both experimental and simulation studies utilized sinusoidal or trapezoidal current waveforms, with a flat-top duration of 0.5 ms for the trapezoids. The pulse duration ($\tau$), defined as the time to ramp from minimum to maximum current, varied from 0.1 to 1.2 ms. In the BG1 coil experiments, waveforms comprised a train of 128 bipolar pulses. To optimize computational efficiency, our simulations employed 16 bipolar pulses, following Davids et al.’s approach [12]. The resultant PNS thresholds were adjusted downwards by approximately 7% to account for the effects of scaling from 16 to 128 bipolar pulses, based on findings by Hebrank et al. in the development of the ’SAFE’ model [7, 12, 18]. They observed that increasing the number of gradient pulses from 16 to 128 reduces the stimulation threshold by about 7%.

## Results

**Fig. 3:**
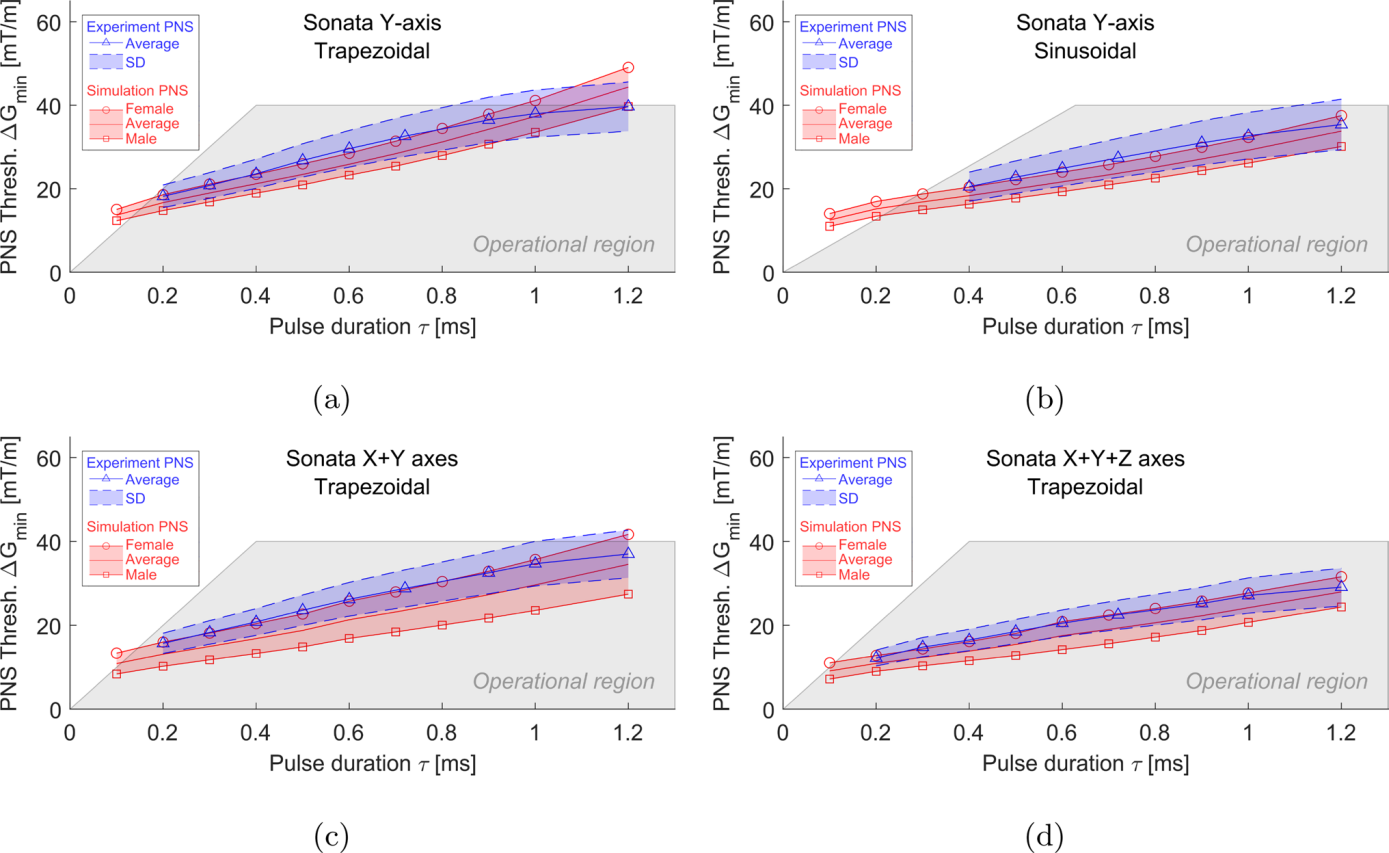
Experimental (blue) and simulated (red) PNS threshold curves with varying pulse durations for trapezoidal and sinusoidal current waveforms applied to BG1 gradient coils in the imaged subject position. The PNS threshold is denoted as the minimum magnetic field gradient strength capable of inducing a PNS

Figure 3 illustrates the experimental and simulated PNS thresholds as they relate to pulse duration ($\tau$) for both trapezoidal and sinusoidal gradient waveforms when applied to BG1 gradient coils. In this figure, the blue curves denote experimental data, whereas the red curves indicate simulation outcomes. The simulated thresholds for the female model were consistently higher than those for the male model at the same pulse duration. In terms of accuracy, compared to the average experimental findings, the female and male models’ average thresholds displayed normalized root-mean-square errors (NRMSE) of 13.6%, 21.3%, 28.6%, and 15.7% for the Y-axis trapezoidal, Y-axis sinusoidal, combined X+Y axes, and combined X+Y+Z axes respectively. However, the experimental results were more closely aligned with the thresholds of the female model, showing NRMSEs of 6.38%, 7.47%, 10%, and 5.27% for these respective cases.

**Fig. 4:**
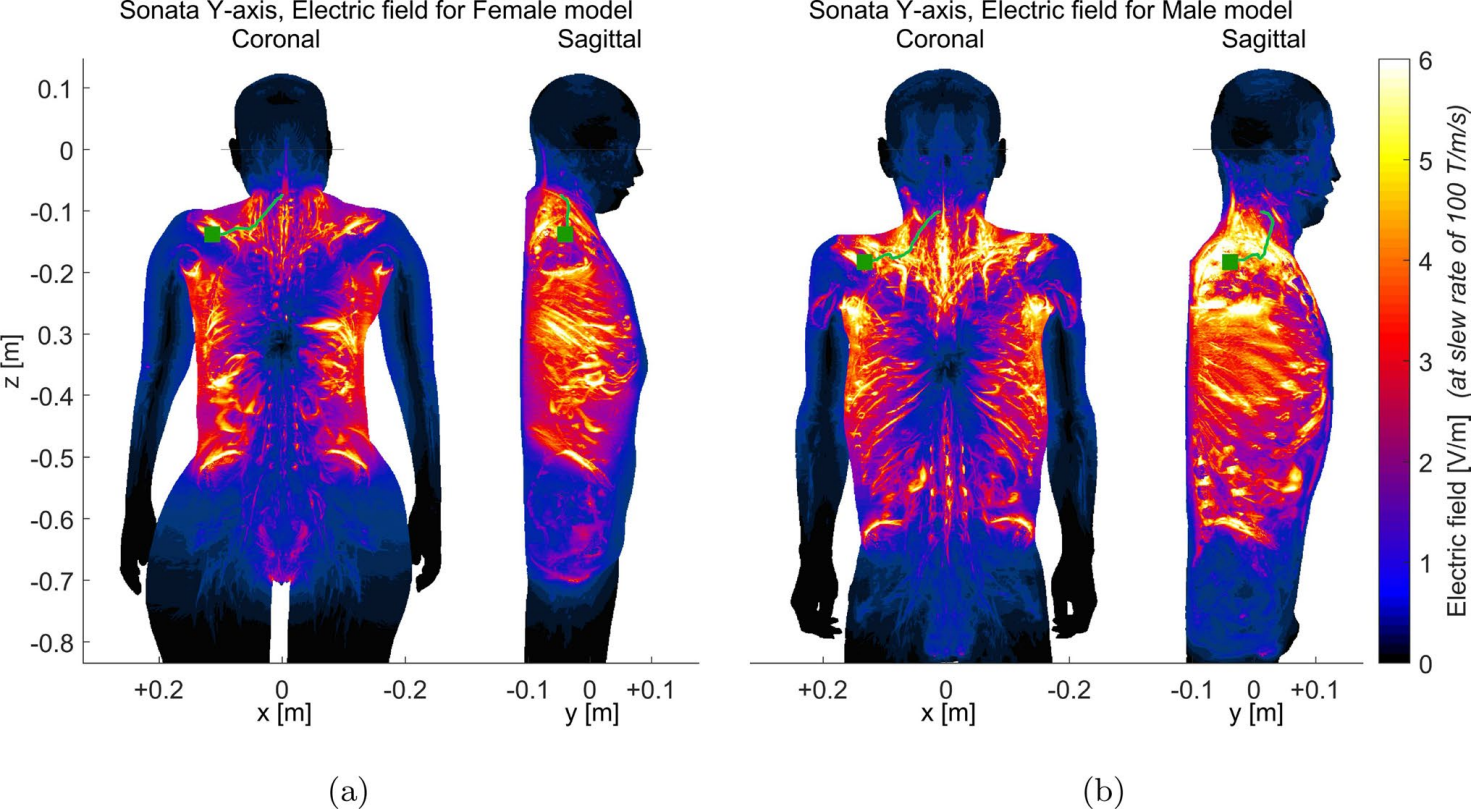
Electric fields (maximum intensity projection) generated by the BG1 y-gradient coil for female (**a**) and male (**b**) models in the imaged subject position, respectively. To enhance visibility of electric field distribution in other body regions, the electric field within the bones was set to 0, as it is typically very high. The stimulated nerves and the first stimulated sites were also indicated with light green curves and dark green squares, respectively

Figure 4 depicts the electric field magnitudes observed in the two human body models positioned as a typical imaged subject, generated by the Gy channel of the BG1 gradient coil. The head of the subject being measured was located within the imaging FOV as shown in Fig. 1a. Areas in proximity to the shoulders, ribs, and hipbones were notable for their high electric field magnitudes. Additionally, the male and female models demonstrated subtle differences in the distribution of these electric fields. Specifically, the shoulder region of the male model exhibited a significantly higher electric field compared to the female model. Conversely, in certain areas around the ribs and the lower body, the female model experienced higher electric fields than the male model. Figure 4 also identifies the stimulated sites in both the female and male human body models for the BG1 Gy gradient coil, with primary stimulation occurring at the suprascapular nerves.

**Fig. 5:**
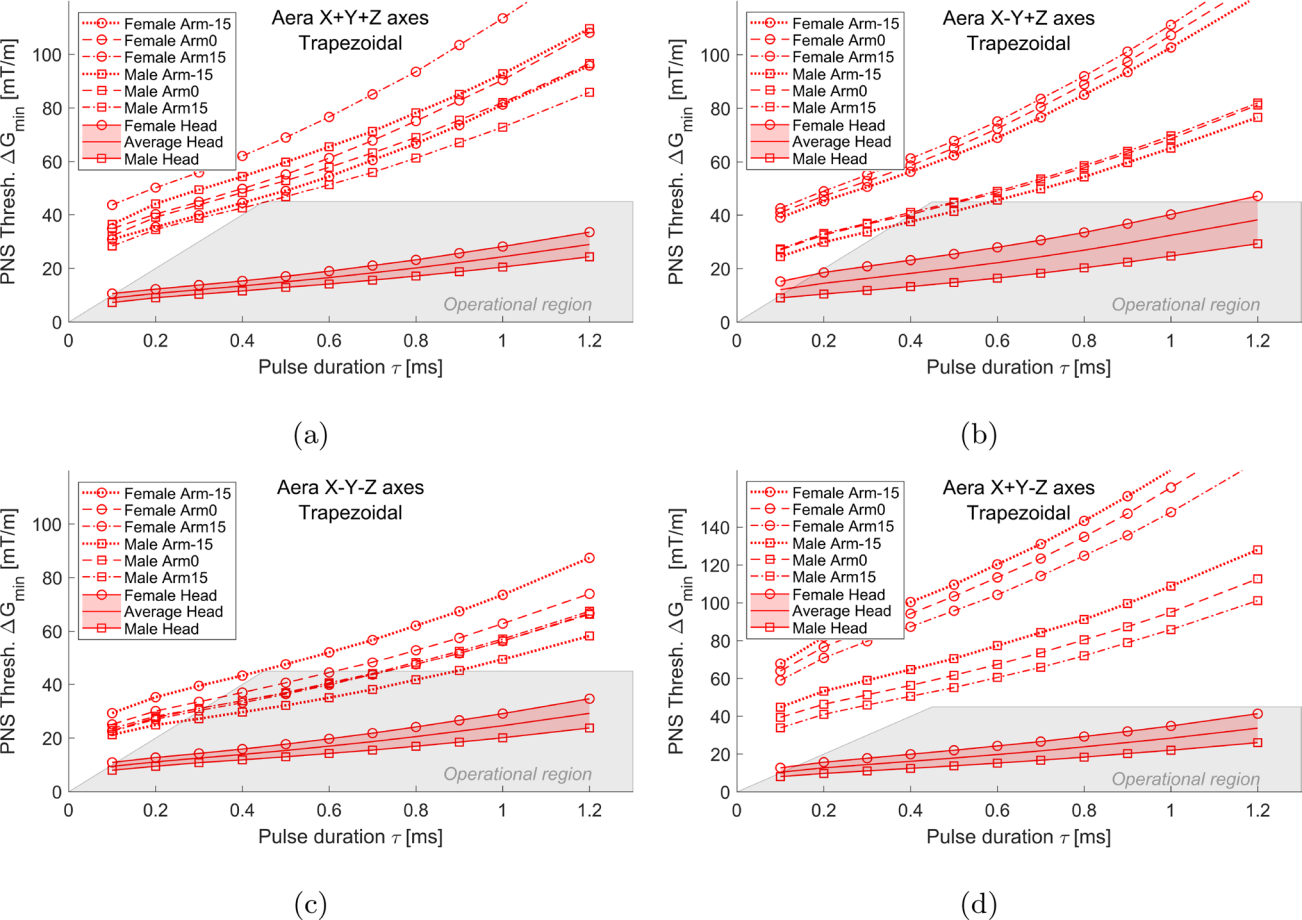
Comparison of PNS thresholds between the imaged subject and interventionalist positions for AG gradient coils. Here, the configurations labeled as ’Female Head,’ ’Average Head,’ and ’Male Head’ correspond to the case of the imaged subject’s position. Various rotational positions of the arm were considered, including Arm0, Arm-15, and Arm15, as shown in Fig. 2. Four different combined axes modes (X+Y+Z, X-Y+Z, X-Y-Z, and X+Y-Z) were investigated, employing three directional gradients generated by AG Gx, Gy, and Gz coils

Figure 5 displays the PNS threshold curves for both the patient position, serving as a reference, and various interventionalist positions with differing arm rotations. In comparison to the imaged subject position, the average thresholds for all interventionalist positions increased by a factor of at least 3.63, 2.69, 2.4, and 4.48 times in the combined axes modes of X+Y+Z, X-Y+Z, X-Y-Z, and X+Y-Z, respectively. Notably, in the X-Y-Z mode for the male model, the lowest among all modes evaluated for the interventionalist, the thresholds were at least double that of the average patient position threshold.

As illustrated in Fig. 5, variations in an interventionalist’s arm rotation induced differing changes in PNS thresholds across various combined axes modes, depending on the body model. For the female interventionalist model, arm rotations of ±15$^{circ}$ from the middle position resulted in PNS threshold fluctuations of up to 25.4%, 4.8%, 18.1%, and 8.1% in the X+Y+Z, X-Y+Z, X-Y-Z, and X+Y-Z modes, respectively. In the case of the male model, similar arm rotations led to threshold variations of up to 13.5%, 9.22%, 15%, and 13% in these respective modes.

The same figure also reveals that the female interventionalist consistently exhibited higher average PNS thresholds than the male counterpart, by at least factors of 1.07, 1.48, 1.14, and 1.61 in the X+Y+Z, X-Y+Z, X-Y-Z, and X+Y-Z modes, respectively. However, an exception was observed in the X-Y-Z mode, where the threshold for a female interventionalist with a 15-degree arm rotation was marginally lower than that of a male with the same arm rotation. Additionally, in the X+Y+Z mode, a −15-degree arm rotation by the female interventionalist led to a decrease in threshold by at least 1.45 times compared to the male interventionalist in the same posture.

Figure 5 also indicates that interventionalists are likely to experience nerve stimulation with various arm rotations in certain combined modes despite hardware constraints imposed by the gradient amplifiers. For instance, in the X-Y-Z mode, the calculated PNS thresholds for male interventionalists, across all three arm rotation scenarios, were within the operational region of the coils when the pulse duration ranged from 0.4 to 0.6 ms. These results can be used to guide experiments aimed at stimulating interventionalists.

Moreover, Fig. 5 highlights that male and female interventionalist thresholds can exhibit divergent trends as arm rotation angles vary, particularly in relation to the combined-axes modes. For instance, in the X-Y+Z mode, both male and female threshold levels increased as the arm rotation shifted from – 15$^{\circ }$ to 0$^{\circ }$, and further to 15$^{\circ }$. Conversely, in the X+Y-Z mode, threshold levels decreased with these angle changes for both models. However, in the X-Y-Z mode, female thresholds decreased while male thresholds increased with the change in arm angle.

**Fig. 6:**
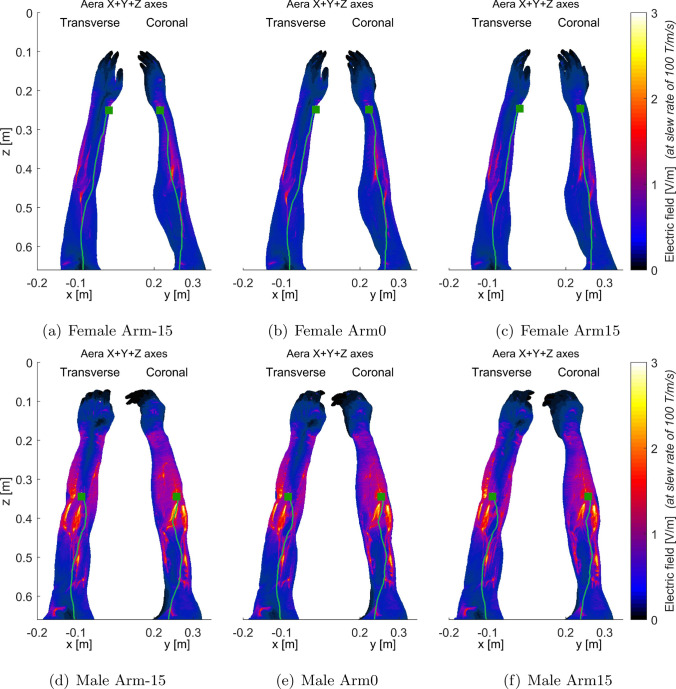
Electric fields (maximum intensity projection) generated by the combined X+Y+Z gradient of AG gradient coils for female (**a**–$^{\circ }$) and male (**d**–$^{\circ }$) models, respectively. The first PNS stimulated sites and the located neuron were identified by dark green squares and light green curves, respectively. Since the first stimulated sites were in the arm regions, only the electric fields in those regions were presented. To better show the electric field distribution in other parts of the arm, the electric field in the bones was set to 0 since that in the bones is usually very high

**Fig. 7:**
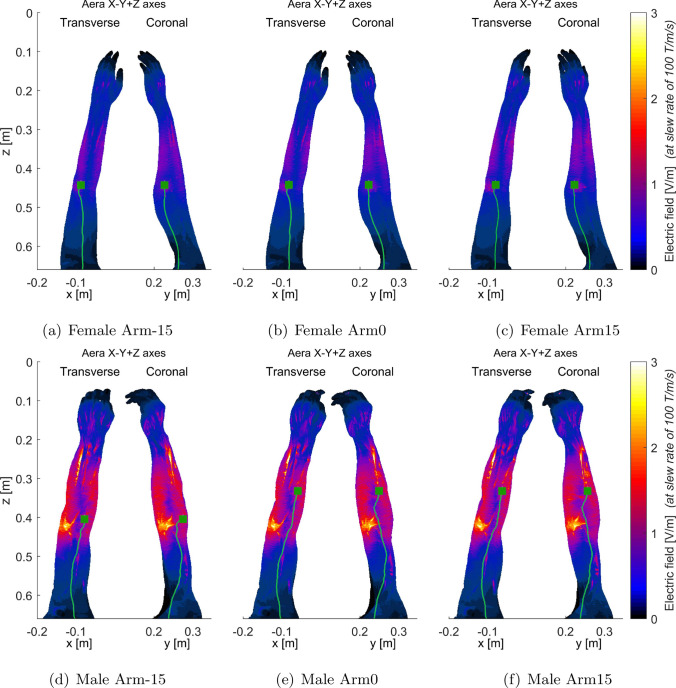
Electric fields (maximum intensity projection) generated by the combined X-Y+Z gradient of AG gradient coils for female (**a**–**c**) and male (**d**–**f**) arm models, respectively. To better show the electric field distribution in other parts of the body, the electric field in the bones was set to 0 since that in the bones is usually very high

**Fig. 8:**
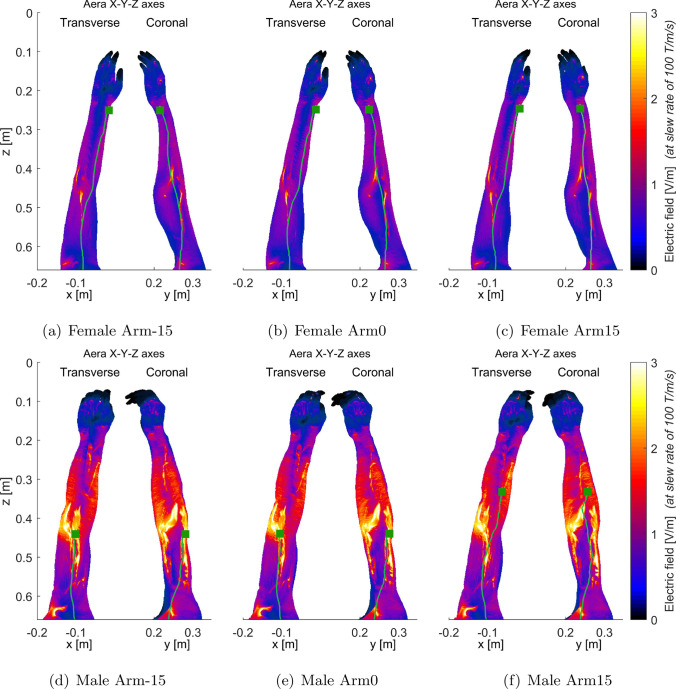
Electric fields (maximum intensity projection) generated by the combined X-Y-Z gradient of AG gradient coils for female arm models (**a**–**c**) and male arm models (**d**–**f**) as illustrated in (Fig. 2). To enhance the visualization of electric field distribution in other body parts, the electric field within the bones was set to 0 as it is typically very high. The stimulated nerves and the first stimulated sites were indicated with light green curves and dark green squares, respectively

**Fig. 9:**
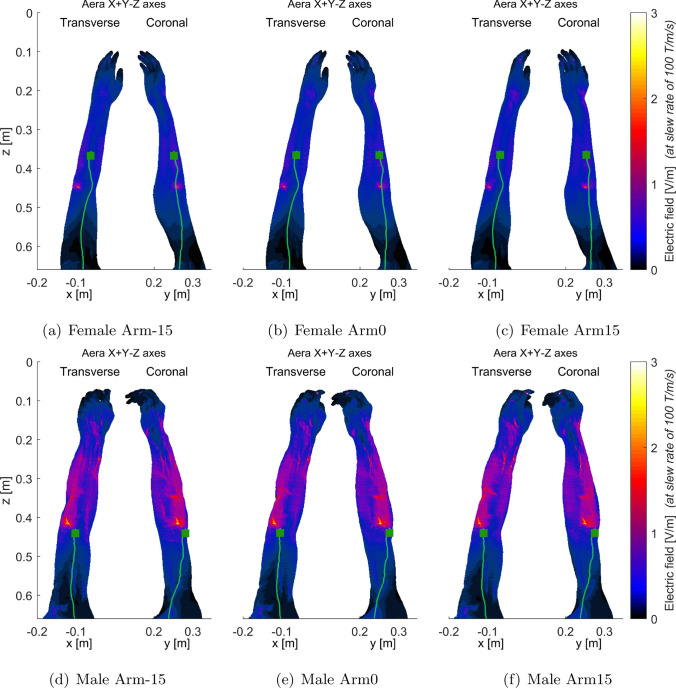
Electric fields (maximum intensity projection) generated by the combined X+Y-Z gradient of AG gradient coils for female (**a**–**c**) and male (**d**–**f**) arm models, respectively. To better show the electric field distribution in other parts of the body, the electric field in the bones was set to 0 since that in the bones is usually very high

Figures 6, 7, 8, and 9 present the electric field distributions corresponding to the X+Y+Z, X-Y+Z, X-Y-Z, and X+Y-Z combined gradient modes, respectively. Notably, the X-Y-Z combined gradient mode is characterized by a comparatively higher electric field distribution than the other three combined gradient modes.

Figures 6, 7, 8, and 9 additionally illustrate the primary PNS sites, marked by dark gray squares, along the nerve tracks in the arm regions for the X+Y+Z, X-Y+Z, X-Y-Z, and X+Y-Z combined gradient modes, respectively. These figures indicate that the location of stimulated sites varies between male and female models within the same gradient mode. For instance, in the X+Y+Z mode, the stimulation site for a female interventionalist is typically near the wrist, while for a male, it is closer to the elbow as depicted in Fig. 6. Additionally, the position of these stimulation sites can be influenced by arm movements as demonstrated in Fig. 8e, f.

## Discussion

This study represents the first study of PNS safety for individuals in close proximity to an MRI system, employing two adjustable human body models in various arm rotation scenarios. To the best knowledge of the authors, the safety for an interventionalist during an interventional procedure has not been assessed, before. As depicted in Fig. 5, arm rotations of a standing model induced up to a 25.4% variation in threshold levels compared to the model’s central position across the four combined-axes modes. Notably, the positions assumed by interventionalists exhibited a minimum 2.4-fold increase in average PNS thresholds relative to those of the subjects imaged with the head at iso-center as illustrated in Fig. 5. Consequently, it can be inferred that ensuring the imaged subject’s safety likely guarantees that the PNS thresholds for the interventionalist are not exceeded.

It is worth noting that our calculations assume a scenario in which patients and interventionalists are separated and do not come into contact. Under these conditions, the electric field distributions within the patients and interventionalists do not affect each other. However, interactions between patients and interventionalists, such as needle placements, may occur during interventional procedures. Ensuring PNS safety in these situations is the topic of future research.

Another point worth mentioning is that this study provides an initial assessment of PNS safety for interventionalists as only three different arm rotation positions were examined for each human model. As shown in Fig. 2, the overall spatial position of the arm remained largely unchanged. This chosen arm position was intended to represent a broader range of individuals. In interventional procedures, various treatments, including radiofrequency ablation [19], cryotherapy [20], and other needle-guided interventions [21], are used to address cancers such as those of the liver, lung, and kidney. A common feature of these procedures is that the target organ is typically positioned near the iso-center of the imaging system. Additionally, treatments are often performed in a posture where instruments or devices are inserted or controlled from above, with the interventionalist’s arm commonly positioned above the patient’s torso to facilitate precise targeting and maneuvering. Bearing in mind the limitations of the MR system length and the arm reach range, we assume the interventionalist’s hand to be positioned above the isocenter somewhat shifted toward the foot end of the scanner with the arm and shoulder nearly touching the upper part of the inner cover of the bore. According to human body statistics [22, 23], the half-thickness of a male torso at the 97.5th percentile is 0.136 m. To account for a wider range of individuals, the arm’s y-direction position was set at approximately 0.15 m, with the arm positioned closer to the scanner’s inner wall, and therefore also closer to the gradient coils. Here, we assumed that as the arm approaches the coil, a stronger electric field may be generated within the arm, potentially resulting in a lower PNS threshold. Based on this assumption, the current arm position might represent a nearly worst-case scenario. Verifying this assumption and investigating how changes in the arm’s position relative to the gradient coil affect the PNS threshold will be the focus of our future research.

The decision to utilize both female and male models is based on Davids et al.’s [10] recommendation for gender representation. According to Davids et al., the normalized root-mean-square errors (NRMSE) in average thresholds between simulated and experimental outcomes were below 5.6% for BG1 gradient coils when employing the Zygote female and male models (Zygote Media Group, American Fork, UT). In contrast, models from the IT’IS foundation yielded NRMSEs as high as 28.6%, as shown in Fig. 3. Despite this discrepancy in NRMSE, PNS thresholds for the male model were generally lower than for the female model, and thresholds linearly correlated with pulse duration of the waveforms. Additionally, the initial stimulation sites were consistent for the BG1 coils. These parallel findings imply that the NRMSE variances are predominantly due to differences between the Zygote and IT’IS foundation models. The human body models used in our study also contribute to diversifying the predictions of PNS thresholds. Moreover, these models represent the sole available options for simulating potential PNS effects due to arm rotations.

As indicated in Fig. 5, the PNS thresholds for male and female interventionalists might exhibit distinct patterns across various combined-axes modes as arm rotation angles vary. This variation in trends could be attributed to the differences in primary PNS sites between male and female models. Figures 6, 7, 8, and 9 illustrate that the electric field distributions surrounding these PNS sites differ, potentially leading to variable PNS threshold levels.

It is worth mentioning that the simulated electric fields, as shown in Figs. 6, 7, 8, and 9, exceed the reference levels and ELVs generally adopted for worker safety [24–27]. Employers and workers should make efforts to reduce their exposure when performing procedures similar to those studied in these simulations.

One limitation of our PNS simulation is the simplification for fiber diameters of peripheral nerves in different body regions. Actually, each nerve consists of many fibers and has a distribution of fiber diameters. For instance, fiber diameters in the human median nerve range from 3.1 to 21.4 $\mu$m [14, 28]. Moreover, detailed investigations of fiber diameter distributions have mainly focused on a limited number of major nerve tracks, such as the sciatic, radial and median nerves, utilizing methods like nerve conduction studies [29] or tissue excisions. In this study, we utilized motor and sensory nerves with fiber diameters of 20 $\upmu$m and 12 $\upmu$m, respectively. This choice is based on the work of Davids et al. (2019), who used these specific diameters and found that their simulation results closely matched the corresponding experimental data.

It is important to note that our reliance on human models has its limitations, particularly due to incomplete nerve atlases. This gap could potentially result in an overestimation of PNS thresholds. Fortunately, the employed models encompass most of the larger peripheral nerves, which are more prone to excitation than their smaller counterparts [30]. A viable approach to validate these findings would be conducting PNS experiments in the standing positions typically used by interventionalists.

At a Siemens Aera scanner at our medical center, preliminary PNS experiments were conducted with two male volunteers in standing positions, utilizing various arm positions. The sequence, consisting of 128 bipolar pulses with a trapezoidal waveform, was generated using Pulseq [31]. The current applied to the Aera gradient coils was constrained by PNS limitations and was set to the maximum using Pulseq. The experiments were performed in X-Y-Z combined-axes modes, with pulse durations ranging from 0.4 to 0.6 ms and gradient amplitudes up to 22 mT/m. Both volunteers reported no PNS, tending to suggest that PNS is unlikely to occur in the body of the interventionalist if the safety limits for the imaged subject are enforced for this specific gradient system. More statistically significant PNS experiments involving a larger number of participants are planned for future research endeavors.

Another point worth noting is that the current research on PNS for radiologists is still limited to the Aera gradient coils. Future research topics will include the corresponding simulations and experimental studies on other high-performance gradient coils, especially new gradient coils designed to control PNS [32] or electric fields [33]. These new types of coils may increase the PNS threshold in certain imaging positions, such as head imaging, but at the same time, they may decrease the PNS threshold in other imaging positions, such as abdominal imaging [34]. Whether these coils will make healthcare professional more susceptible to stimulation is another research question we plan to explore.

## Conclusion

Two posable human body models were used to predict PNS thresholds for a typical standing position of a healthcare professional doing an MRI-guided intervention. The preliminary results suggest that the interventionalist is unlikely to experience PNS before stimulating the patient, regardless the studied arm rotations. Arm rotations have an strong influence on the site of stimulation in the arm. In addition, it should be noted that the study including the interventionalist’s position was conducted using only one specific gradient system..

## Data Availability

The data that support the findings of this study are not openly available due to reasons of sensitivity and are available from the corresponding author upon reasonable request. Data are located in controlled access data storage at University medical center Freiburg.
